# Photobiomodulation Mitigates Diabetes-Induced Retinopathy by Direct and Indirect Mechanisms: Evidence from Intervention Studies in Pigmented Mice

**DOI:** 10.1371/journal.pone.0139003

**Published:** 2015-10-01

**Authors:** Alexandra Saliba, Yunpeng Du, Haitao Liu, Shyam Patel, Robin Roberts, Bruce A. Berkowitz, Timothy S. Kern

**Affiliations:** 1 Case Western Reserve University, Cleveland, Ohio, United States of America; 2 Catholic University of Brasilia, Brasilia, Brazil; 3 Department of Anatomy and Cell Biology, Wayne State University, Detroit, Michigan, United States of America; 4 Department of Ophthalmology, Wayne State University, Detroit, Michigan, United States of America; 5 Cleveland Veteran’s Affairs Medical Center, Research Service 151, Cleveland, Ohio, United States of America; University of Melbourne, AUSTRALIA

## Abstract

**Objective:**

Daily application of far-red light from the onset of diabetes mitigated diabetes-induced abnormalities in retinas of albino rats. Here, we test the hypothesis that photobiomodulation (PBM) is effective in diabetic, pigmented mice, even when delayed until weeks after onset of diabetes. Direct and indirect effects of PBM on the retina also were studied.

**Methods:**

Diabetes was induced in C57Bl/6J mice using streptozotocin. Some diabetics were exposed to PBM therapy (4 min/day; 670 nm) daily. In one study, mice were diabetic for 4 weeks before initiation of PBM for an additional 10 weeks. Retinal oxidative stress, inflammation, and retinal function were measured. In some mice, heads were covered with a lead shield during PBM to prevent direct illumination of the eye, or animals were treated with an inhibitor of heme oxygenase-1. In a second study, PBM was initiated immediately after onset of diabetes, and administered daily for 2 months. These mice were examined using manganese-enhanced MRI to assess effects of PBM on transretinal calcium channel function *in vivo*.

**Results:**

PBM intervention improved diabetes-induced changes in superoxide generation, leukostasis, expression of ICAM-1, and visual performance. PBM acted in part remotely from the retina because the beneficial effects were achieved even with the head shielded from the light therapy, and because leukocyte-mediated cytotoxicity of retinal endothelial cells was less in diabetics treated with PBM. SnPP+PBM significantly reduced iNOS expression compared to PBM alone, but significantly exacerbated leukostasis. In study 2, PBM largely mitigated diabetes-induced retinal calcium channel dysfunction in all retinal layers.

**Conclusions:**

PBM induces retinal protection against abnormalities induced by diabetes in pigmented animals, and even as an intervention. Beneficial effects on the retina likely are mediated by both direct and indirect mechanisms. PBM is a novel non-pharmacologic treatment strategy to inhibit early changes of diabetic retinopathy.

## Introduction

Diabetes is a major cause of visual impairment, and there is considerable clinical and research interest in diabetic retinopathy. Proven therapeutic approaches, such as good glycemic control, high energy laser photocoagulation, or intravitreal injections of anti-VEGF therapies or triamcinolone, are invasive, damaging, or require direct involvement by health-care professionals, and not all patients respond to these approaches. Supplemental therapeutic approaches are needed.

Photobiomodulation (PBM) is the application of low-level light that has a biological effect, such as to relieve pain or heal wounds. Numerous studies have shown that light in the far-red to near-infrared region of the spectrum (630–1000 nm) can have beneficial effects *in vitro* and *in vivo* to heal existing tissue damage and to inhibit the development of tissue pathology. Medical PBM using coherent (lasers) or noncoherent (Light Emitting Diodes; LEDs) light has been found to have beneficial effects in a variety of conditions, including accelerated healing of wounds and ulcers, cardiac ischemia, stroke, Parkinson’s disease, and optic nerve degeneration [1–12]. Studies related to the retina likewise have demonstrated that the low intensity light treatment mitigates pathology in retinal degeneration models [4,13–16], and recently, also in diabetic retinopathy [17,18]. Our previous study in diabetic albino rats showed that whole-body exposure to far-red light (670 nm) for only 4 minutes per day from the onset of diabetes mitigated abnormalities that are believed to contribute to diabetic retinopathy, including increased generation of superoxide, induction of a local pro-inflammatory environment, and dysfunction or degeneration of retinal neurons [18].

The purpose of the present study was to extend those studies to determine if PBM would have similar beneficial effects under the following different conditions: *(i)* in another species (mice), *(ii)* in the presence of heavy pigmentation (C57Bl/6J), *(iii)* as an intervention therapy, *(iv)* when direct exposure of the eyes to the PBM was blocked, and *(v)* when activity of the antioxidant enzyme, heme oxygenase 1 (HO-1), was inhibited. Our results suggest that the PBM has both neuronal and vascular beneficial effects on pigmented diabetic mice, and that this effect is mediated at least in part systemically.

## Materials and Methods

This study was performed in strict accordance with the National Institutes of Health Guide for the Care and Use of Laboratory Animals, the Association for Research in Vision and Ophthalmology Statement for the Use of Animals in Ophthalmic and Vision Research, and with authorization of the Institutional Animal and Care Use Committee (IACUC) at Case Western Reserve University and Wayne State University. Animals were housed and maintained in normal 12h:12h light-dark cycle laboratory lighting.

### Animals

Male C57BL/6J mice were obtained from the Jackson Laboratory, and were housed in ventilated microisolator with free access to water and food. Diabetes was induced at 2–3 months of age by intraperitoneal injection of a freshly prepared solution of streptozotocin in citrate buffer (55 mg/Kg of body weight for five consecutive days). Insulin was given as 0–0.2 units subcutaneously between 0–3 times a week to inhibit weight loss, while still allowing hyperglycemia. To allow the animals to stabilize somewhat after induction of diabetes, blood glucose concentration was not measured until at least 7 days after the final administration of streptozotocin. Blood glucose was determined with a portable glucose meter, using blood collected from the tail vein under nonfasting conditions. The onset of diabetes was defined as three consecutive measures of blood glucose over than 275 mg/dl. HbA1c was measured as reported previously (Study 1 [19,20]; Study 2 [21].

There were two parts to this work. In the first, five groups with n = 12 animals per group were assigned as: *(i)* non-diabetic controls, *(ii)* diabetic controls, *(iii)* diabetic exposed to PBM starting 4 weeks after the induction of diabetes, *(iv)* diabetic exposed to PBM while the head was shielded from the light by a lead covering, and *(v)* diabetic treated with a heme oxygenase-1 (HO-1) inhibitor (tin protoporphyrin, SnPP; Frontier Scientific Inc, Logan, UT) [22], starting 4 weeks after the induction of diabetes. All animals were euthanized at 14 weeks of diabetes (5–6 months of age). In the second study, three groups were studied: (1) non-diabetic untreated control (n = 9), (2) diabetic untreated control (n = 3), and (3) diabetic treated with PBM from the onset of diabetes (n = 5). These animals were humanely euthanized at 8 weeks of diabetes.

### Photobiomodulation

The far-red light was generated by LEDs (SpectraLife™; Quantum Devices, WI). This device was determined to deliver 670nm light at a power of 20.25 mW/cm^2^ at the 2–3 cm distance used between the device and the animal (measured with Spectro-radiometer; specbos 1211UV, Dataoptics, Inc, Ypsilanti MI). The mice were exposed to this radiation for 240 sec each day for the 10 weeks. The daily radiant exposure was thus 240 x 20.25 = 4860 mJ/cm^2^ (or ~5 J/cm^2^). In study 1, treated mice were placed in a DecapiCone™ restrainer bag (Braintree Scientific, Braintree, MA), and then in an open-top polypropylene holder using a Velcro strap to secure the animals. To ensure that the polypropylene bag would not impair passage of the red light, transmittance through the bag was tested; compared to the air, one layer of the plastic used to restrain the animals absorbed 8.2% of the light (0.082 Abs) at 670 nm. Intervention with PBM treatment was started four weeks after the onset of diabetes, and was continued for ten additional weeks. To minimize handling, animals in study 2 were allowed free movement in a small space during PBM. PBM was started immediately after confirmation of diabetes in this experimental study.

### Retinal Superoxide

Fresh retinas from animals were analyzed for superoxide production as previously described [18,23,24]. Briefly, retinas were placed in 0.2 ml of Krebs/HEPES buffer and allowed to equilibrate in the dark at 37°C under 95% O_2_/5% CO_2_ conditions for 20 min. To each tube, 0.5 mM lucigenin (Sigma Chemical Company, St. Louis, MO) was added and incubated for an additional 10 minutes before having the photon emission detected by a luminometer (Analytical Luminescence Laboratory, San Diego, CA). Retinal protein was quantified (Bio-Rad), and the luminescence expressed per mg of protein.

### HO-1 inhibition

Activity of HO-1 was inhibited systemically with SnPP in one of the diabetic groups getting the PBM treatment. The SnPP was freshly made dissolved in 0.2 N NaOH, adjusted to physiological pH 7.4 with 1N HCl, and protected from light. Mice received 100 μmol/kg once per week by intraperitoneal injection. This dose of SnPP has been reported to inhibit HO-1 activity in vivo [25].

### Leukostasis

Blood was removed from the vasculature of anesthetized animals by perfusion with phosphate-buffered saline (PBS; 10 mM phosphate buffer, 2.7 mM potassium chloride and 137 mM sodium chloride, pH 7.4; Sigma-Aldrich, St. Louis, MO) via a heart catheter. Animals were then perfused with fluorescein-coupled concanavalin A lectin (20 μg/ml in PBS; Vector Laboratories, Burlingame, CA), as described previously [20,23,26]. Flat-mounted retinas were imaged via fluorescence microscopy, and the number of leukocytes adherent to the vascular wall was counted.

### Visual Function

The virtual optokinetic system (OptoMetry; CerebralMechanics, Inc,) was used as described previously [19,27]. At 14 weeks of diabetes, mice were allowed to move freely on the platform of the visual OptoMotor system while the visual stimulus was projected around the platform. Spatial frequency threshold was measured at maximum contrast. For contrast sensitivity, only a single value (0.064 c/d; the maximal point in a full contrast sensitivity curve) was measured. These tests are psychophysical measures that assess function of both retinal and central visual pathways. Each measurement was repeated several times to evaluate the reproducibility of responses. The grader was masked with respect to the animals’ experimental group.

### Immunoblots

Immunoblots of ICAM-1 (intercellular adhesion molecule-1; 1:500 dilution, R&D Systems, MN), iNOS (1:1000 dilution, Santa Cruz, CA) and HO-1 (1:1000 dilution, Cell Signaling Technology, Inc., Danvers, MA) were performed using the respective antibodies. The retina or cells was isolated and placed into 80 μl of lysis buffer supplemented with protease inhibitor (1:1000 dilution), sonicated and centrifuged. The supernatant was collected and each sample containing the same amount of protein was fractionated by SDS-PAGE and electroblotted to PVDF membranes. After blocking nonspecific binding with 5% BSA, the membranes were incubated with rabbit polyclonal antibodies followed by incubation with antibody against rabbit IgG. Densities were normalized to that of ß-actin in the same lane (1:3000 dilution, Abcam, Inc., Cambridge, MA).

### Leukocyte-mediated killing of retinal endothelial cells [20,23,28]

Transformed mouse retinal endothelial cells (mREC; generated from Immortomice [29]) were grown in DMEM containing 10% FBS and 5 or 25 mM glucose. Cells were cultured at 37°C in 5% CO_2_ and 95% air, and the media was changed every other day for 5 days. When mRECs were 80% confluent (approx. 500,000 cells), leukocytes (100,000; enriched from blood with RBC lysis buffer) from animals in the nondiabetic, diabetic, and diabetic treated with PBM groups were added to the mREC and incubated for 24 hrs [23]. After incubation, the leukocytes were carefully removed by gentle washing, and viability of remaining retinal endothelial cells was measured by trypan blue exclusion. Briefly, an aliquot of the endothelial cell suspension was diluted 1:1 (vol/vol) with 0.1% trypan blue, and the cells were counted with a hemocytometer. Cell death was reported as the percentage of blue-stained cells (dead cells) of the total number of cells. Approximately 200–400 cells were counted in each sample.

### MEMRI

MEMRI is the imaging modality of choice for non-invasively studying the function of voltage-gated Ca(2+) channels on excitable cells, such as rod cell L-type calcium channels [30,31]. The MEMRI procedure in mice has been described previously [32–35]. MEMRI data from the central retinal (± 1 mm from the center of the optic nerve) were analyzed using the region-of-interest following our prior procedure [32–35]. The resolution obtained in the central retina is sufficient for extracting meaningful layer-specific anatomical and functional data, as previously discussed [36,37]. In the present studies, whole-retinal thicknesses in nondiabetic mice was ~ 220 μm studied with an axial pixel size of 25 μm [36]. Thus, the spatial uncertainty is ~ ½ pixel thick (~12.5 μm) [36]. We rely on the well-defined laminar structure of the retina to distinguish, for example, inner retinal uptake from outer retinal uptake.

### Statistical Analyses

Data were expressed as means ±SD, unless otherwise noted. Groups were compared using analysis-of-variance followed by Fischer’s test, with p ≤ 0.05 being considered statistically significant. MEMRI data (mean ± SEM) were analyzed using a generalized estimating equation (GEE) approach which performs a general linear regression analysis using contiguous locations or measurements in each subject and accounts for the within-subject correlation between contiguous locations or measurements [37,38]. Comparisons of MEMRI data between groups were performed using individual t-tests at different locations of the intraretinal profiles; those selected regions identified from the t-tests as significant were then analyzed using GEE [37,38]

## Results

### Animals

After injection with STZ, the presence of diabetes was confirmed based on blood glucose levels and body weight. Over the weeks of diabetes, the mean blood glucose level, HbA1c and body weight were significantly different between STZ-treated mice and control mice (Tables 1 and 2). PBM treatment had no significant effect on body weight, non-fasting blood glucose or HbA1c compared to the other diabetic groups.

**Table 1:** Animal Data. Average body weight, non-fasting blood glucose, and HbA1c of nondiabetics (N), diabetics (D), diabetics receiving PBM therapy (D+ PBM), diabetics receiving PBM therapy with head shield (D+ PBM + HS), and diabetics receiving PBM therapy and SnPP treatment (S+ PBM+ SnPP).

	N	D	D+ PBM*	D+ PBM+ HS*	D+ PBM+ SnPP*
**Body Weight (g)**	36 ± 4	29 ± 3**	26 ± 2	28 ± 2	27 ± 1
**Nonfasting blood glucose (mg/dL)**	163 ± 20	544 ± 54**	547 ± 40	526 ± 65	508 ± 49
**HbA1c (%)**	3.2 ± 0.2	8.2 ± 0.6**	8.3 ± 0.5	8.4 ± 0.7	8.5 ± 0.4

* PBM initiated 4 weeks after induction of diabetes, and continued for an additional 10 weeks (total, 14 weeks)

** p<0.001 compared to N; there are no significant differences among diabetic groups

**Table 2:** Average body weight and HbA1c of nondiabetics (N), diabetics (D), and diabetics receiving PBM therapy (D+ PBM) in Trial 2.

	N	D	D+PBM*
**Body Weight (g)**	29 ± 1	25 ± 1**	24 ± 1
**HbA1c (%)**	5.9 ± 0.3	11.7 ± 0.6**	11.6 ± 0.6

* *PBM initiated from the onset of diabetes*, *and continued for a total of 8 weeks*

** *p<0*.*001 compared to N; there are no significant differences among diabetic groups*

### Study 1

#### Intervention with PBM mitigated diabetes-induced molecular abnormalities in retinas from pigmented mice

**Fig 1:**
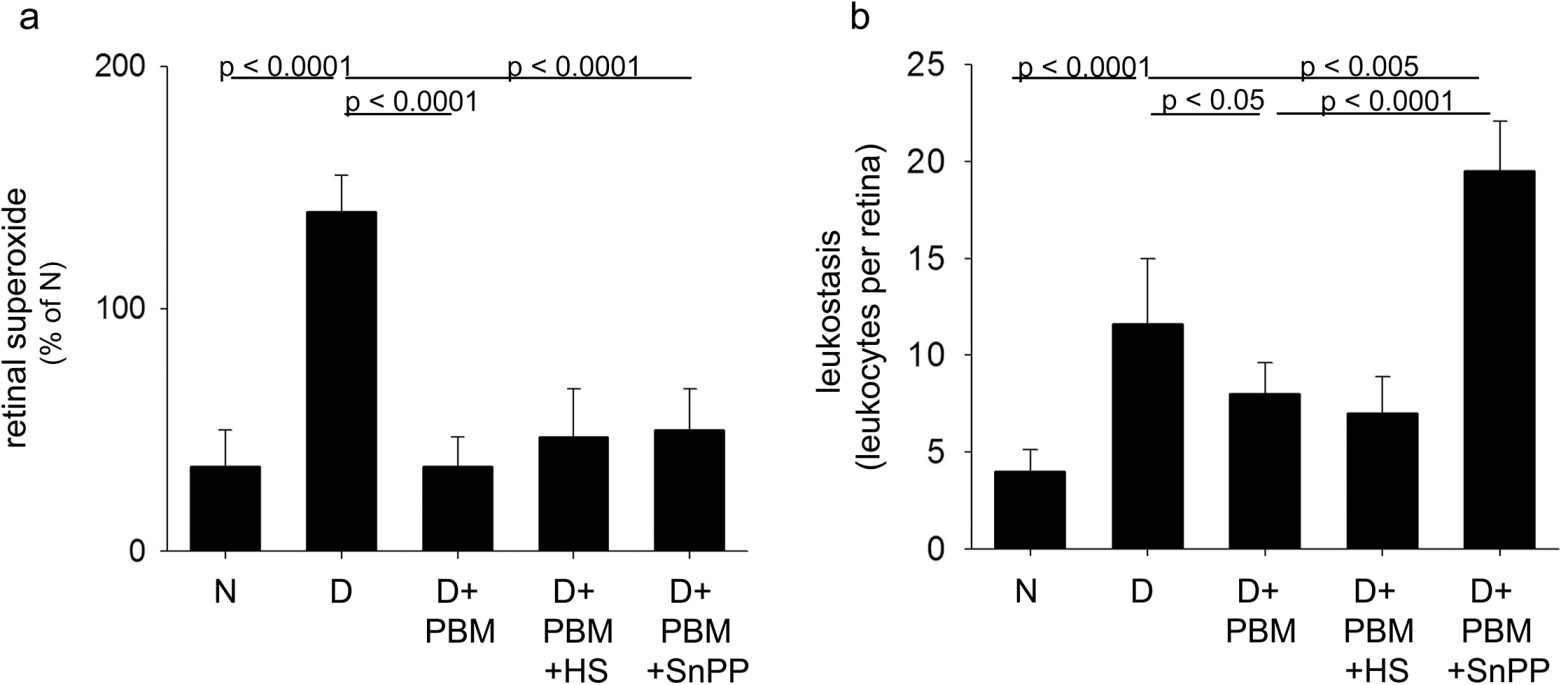
Intervention with PBM mitigated diabetes-induced (a) generation of superoxide by retina and (b) leukostasis in the retinal vasculature. Some diabetic mice treated with PBM had their head shielded from the light by a lead shield (PBM + HS) or were concurrently treated with the HO-1 mitigator, SnPP (PBM + SnPP). Total duration of diabetes was 14 weeks, but PBM and HS or SnPP were applied only for the last 10 weeks of that interval. N, nondiabetic; D, diabetic; D+PBM, diabetics receiving PBM therapy. Horizontal lines above the figure indicate significant differences; no line indicates lack of statistical significance. n = 4–6 per group.

**Fig 2:**
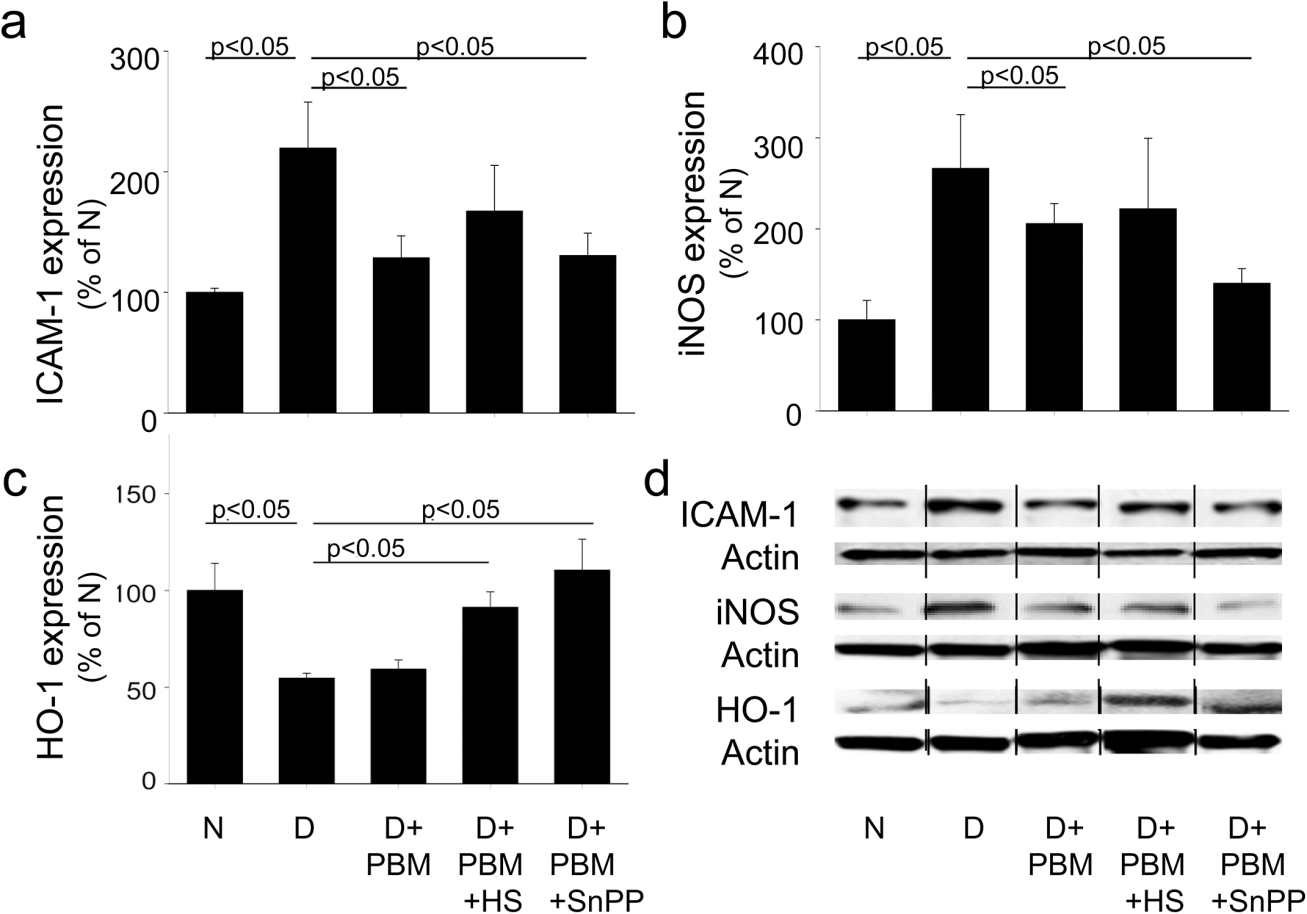
Effect of PBM intervention on retinal (a) ICAM-1, (b) iNOS and (c) HO-1 in diabetic mice. Intervention with PBM significantly reversed the diabetes-induced induction of retinal ICAM-1, whereas effects on iNOS and HO-1 expression were less marked or absent. Use of the head shield (PBM + HS) or inhibiting HO-1 with SnPP (PBM + SnPP) did not affect the effect of PBM on expression of ICAM-1 significantly, but SnPP did tend to normalize iNOS expression, and both the head shield and SNPP increased HO-1 expression. Representative immunoblots are shown in (c). Total duration of diabetes was 14 weeks, but PBM was applied only for the last 10 weeks of that duration. N, non-diabetic; D, diabetic; D+PBM, mice getting PBM. Horizontal lines above the figure indicate significant differences. n = 4–6 per group.

In wildtype C57Bl/6J mice, diabetes caused metabolic and physiologic abnormalities in the retina, including increased superoxide production, leukostasis (Fig 1), increased expression of ICAM-1 and iNOS, and subnormal expression of HO-1 (Fig 2). Diabetes of 2 months duration also significantly impaired visual function, as assessed from spatial frequency threshold and contrast sensitivity (measured at a single point; 0.064 c/d) (not shown; both p<0.0001). Although initiation of the PBM therapy in these pigmented animals was delayed for 1 month of untreated diabetes, the subsequent intervention with PBM therapy nevertheless significantly mitigated many of these defects. Intervention with daily exposure to the PBM totally inhibited the increase in retinal superoxide, and significantly inhibited diabetes-induced abnormalities in leukostasis, retinal ICAM-1 expression, and spatial frequency threshold (p<0.0001). The light therapy had no significant effect on the diabetes-induced changes in retinal iNOS or HO-1 or defect in contrast sensitivity measured in these samples.

### HO-1

In vitro studies previously showed that incubation of retinal cells in 30 mM glucose caused a significant reduction in HO-1 expression, and that PBM significantly inhibited this reduction [18]. To determine if this effect on HO-1 contributed to the observed beneficial effects of PBM in vivo, we simultaneously administered PBM along with a pharmacologic inhibitor of HO-1 (SnPP) for the 10 weeks with the light treatment. Pharmacologic inhibition of HO-1 in vivo completely prevented the benefits of PBM in the diabetes-induced increase in retinal leukostasis (Fig 1), suggesting that the beneficial effect of PBM on leukocyte adhesion required HO-1. In contrast, inhibition of HO-1 did not counteract the effect of PBM on the diabetes-induced generation of superoxide or expression of ICAM-1, iNOS or HO-1. In fact, inhibition of HO-1 during PBM suppressed the diabetes-induced induction of iNOS expression better than that observed with PBM alone. Surprisingly, both the head shield and SNPP increased HO-1 expression. Administration of SnPP to PBM-treated diabetics had no significant effect on spatial frequency threshold or single point contrast sensitivity compared to animals treated with PBM only (not shown).

### Beneficial effects of PBM are mediated in part via systemic effects

**Fig 3:**
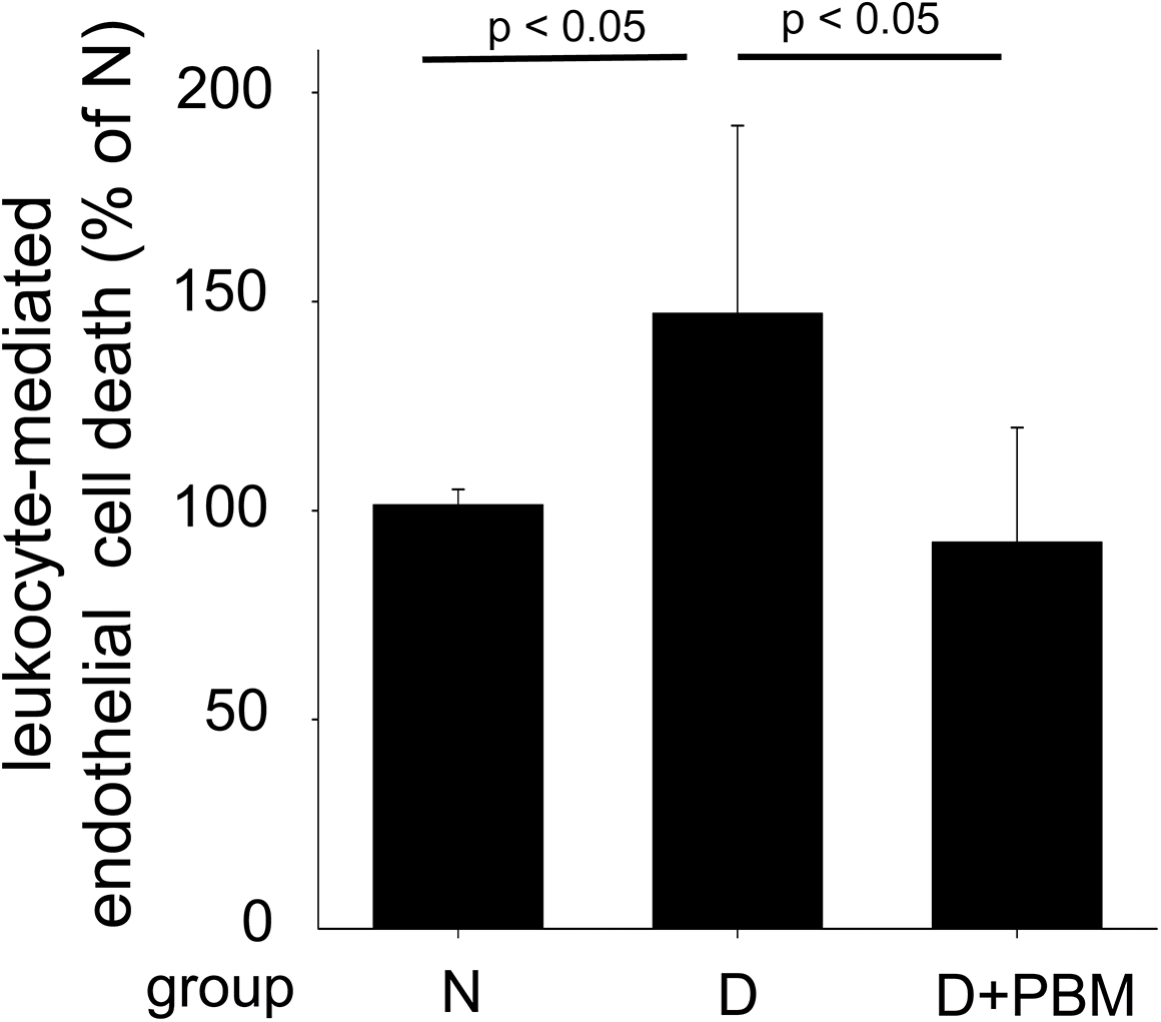
Intervention with PBM mitigates leukocyte-mediated cytotoxicity of endothelial cells caused by leukocytes from diabetic animals. Co-culture of transformed mouse retinal endothelial cells (mREC) with leukocytes from diabetic mice resulted in significantly more dead endothelial cells than when leukocytes were isolated from nondiabetic animals. In contrast, diabetes-induced killing of endothelial cells in the co-culture assay was significantly inhibited when using leukocytes isolated from diabetic animals who had been treated daily with PBM as an intervention therapy. N, non-diabetic; D, diabetic; D+PBM, mice getting PBM as an intervention for the last 10 weeks of diabetes. Horizontal lines above the figure indicate significant differences. n = 3 per group.

To test if some of the observed beneficial effects of PBM on retina were mediated systemically, an opaque (lead) head shield was used to prevent direct irradiation to the eye. Neither retinal superoxide production, leukostasis, iNOS (Figs 1 and 2), nor the tests of visual function differed appreciably between the diabetics getting PBM with or without the head shield, suggesting that the beneficial effects of PBM on the retina in diabetes did not require direct illumination of the head or eyes for these parameters. As an additional test of PBM’s systemic effects, we assessed the ability of PBM to exert beneficial effects on leukocytes flowing in the systemic circulation. We previously have found that leukocytes play a major role in diabetes-induced degeneration of retinal capillaries [23,39]. Consistent with our prior reports, leukocytes from control diabetics (not treated with PBM) caused significantly more endothelial cytotoxicity in co-cultures than did leukocytes from nondiabetic animals (Fig 3). Intervention with PBM for only 4 min per day totally suppressed the diabetes-induced killing of retinal endothelial cells by leukocytes from diabetic mice.

### Study 2

#### PBM mitigated diabetes-induced calcium channel dysfunction across all retinal layers

**Fig 4:**
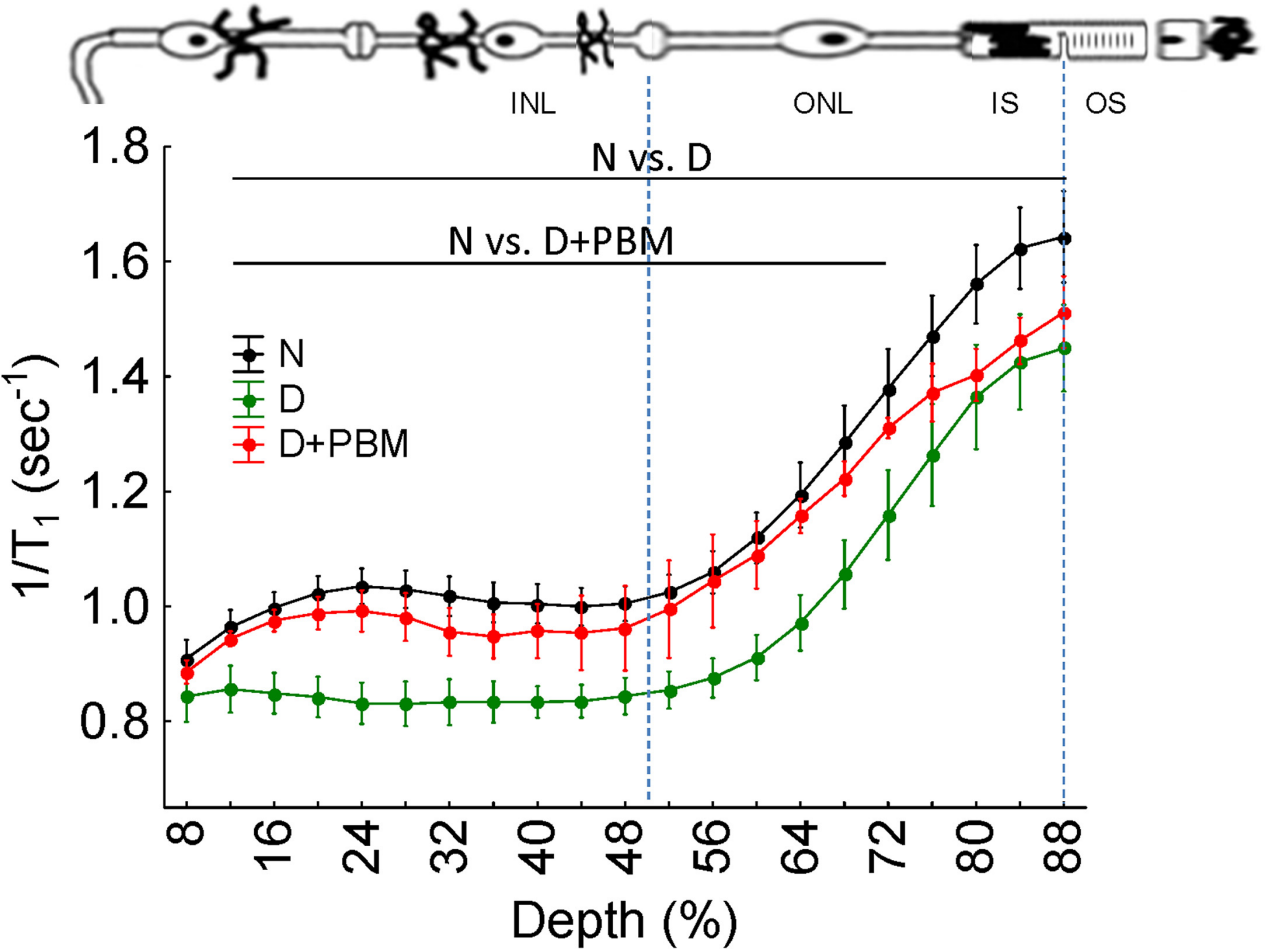
Preventive PBM treatment corrects diabetes-induced calcium channel dysfunction across the retina. Central (± 0.4–1 mm from the optic nerve head) retinal manganese uptake (as evaluated via 1/T1) profiles of A) dark adapted WT mice (closed black circles, n = 9), untreated diabetic mice (closed green circle, n = 5), and PBM treated diabetic mice (closed magenta circle, n = 5). Data are shown as a function of distance from the retina / non-retina borders, where 0% is the vitreous/retina border and 100% is the retina/choroid border. Regions near borders are not shown because these regions likely include some signal from outside the retina (i.e., partial volume averaging with vitreous or choroid/sclera). Lines above profiles indicate retinal regions with statistically significant differences between indicated groups. A simplified schematic of retina is provided to provide best estimates of MEMRI localization in the retina. The high resolution of our MEMRI images (21.9 μm axial resolution [40]) and the well-defined laminar structure of retina allows us to reasonably label uptake at 24–50% depth as the inner nuclear layer (INL), at 50–68% depth as the outer nuclear layer (ONL), at 68–88% depth as the rod inner segment region (IS), and > 88% as the rod outer segment region (OS).

As previously reported [21,33], diabetes reduced manganese uptake across the retina in the dark compared with non-diabetic mice (Fig 4). PBM-treated diabetic mice exhibited largely normal calcium channel function in all layers of the retina, except in the presumptive rod inner segment layer.

## Discussion

PBM is a novel therapeutic approach to mitigate the development of diabetic retinopathy, and preliminary studies in diabetic rodents and patients have shown promise [17,18]. Of note, the therapy is easy to administer, and the beneficial effects were detected at a low amount of daily radiant exposure. We demonstrate herein that the beneficial effects of PBM are mediated at least in part via indirect effects, and occur even if the initiation of therapy is delayed (post-conditioning).

We used a head shield to determine if observed beneficial effects of PBM on the retina required direct illumination of the retina. The diabetic mice that had their heads covered to prevent light from reaching the eyes for the 4 min of illumination nevertheless showed beneficial effects of the PBM treatment with respect to superoxide generation, leukostasis, expression of inflammatory proteins, and visual function. Previously, others [11,12,41] reported that beneficial effects of PBM in a model of Parkinson’s disease were not inhibited by a head shield, likewise suggesting that the neuroprotective effects of PBM in that model were mediated at least partly by indirect (systemic) means. Indirect effects of PBM detected also in other studies include where treatment of one side of the body had beneficial effects also on the untreated side with regard to skin blood flow, temperature in the feet of diabetic patients [42], or healing of skin wounds and burns [43,44]. Previously reported beneficial effects of PBM in kidney and heart [7,8] of diabetic animals are consistent with PBM exerting at least some effects via systemic mediators, but far-red light is known to penetrate deeply into tissues, and thus might directly irradiate those tissues.

The molecular and cellular changes initiated by PBM which mediate observed beneficial effects are under investigation. Mitochondrial cytochrome c oxidase is often considered to be a target of PBM therapy [15,45–47], although a recent study from our group did not find evidence to support this in the retina of diabetic rats [18]. Intriguingly, MEMRI studies showed that PBM did not inhibit diabetes-induced defects in ion movement in the inner segment layer of retinal photoreceptors, the region that contains the majority of retinal mitochondria. Thus our findings provide suggest that PBM exerts beneficial effects that are independent of photoreceptor mitochondria.

In addition, several studies have demonstrated that PBM can activate stem cells to proliferate [48–50]. This might be relevant, since release of stem cells and progenitor cells from bone marrow is impaired in diabetes [51–54], and administration of stem cells to diabetic animals inhibits lesions of the retinopathy [55].

PBM imparts significant anti-oxidant benefits in retinas of diabetic albino rats and in pigmented mice. Whether this is mediated via direct or indirect mechanisms is not known, but it is clear that increased retinal oxidative stress is a major contributor to the pathogenesis of diabetic retinopathy. Recent studies suggest that this oxidative stress is generated in large part by rod cells [24]. Oxidative stress is a known modulator of calcium channel function [56], so we assessed effects of PBM on diabetes-induced alterations in retinal ion movement using MEMRI. Previous studies have shown that the diabetes-induced impairment in intraretinal manganese uptake are corrected by antioxidants (including lipoic acid and targeted overexpression of peroxisomal catalase) or overexpression of the antioxidant enzyme, superoxide dismutase [33,57]. We now show that PBM improves both inner and outer retinal uptake of manganese, consistent with the hypothesis that retinal oxidative stress in diabetes impairs ion channel function, and that mitigation of the ion channel defects by PBM occurs secondary to inhibition of the oxidative stress. How PBM affects calcium channel function is unclear at present.

HO-1 is a highly inducible enzyme that mediates cytoprotective responses to toxic insults, including inflammation and oxidative stress. The enzyme or its products have important cardiovascular protective effects, such as vasodilator, anti-inflammatory, antihypertensive, antioxidant and anti-apoptotic actions [58–61]. To determine if HO-1 played a role in the observed beneficial effects of PBM in retinas of diabetic animals, some diabetics treated with PBM were also given the HO-1 inhibitor, SnPP, with the expectation that parameters made worse by the inclusion of the HO-1 inhibitor would indicate possible roles of HO-1 in beneficial effects of PBM. Compared to diabetics with PBM alone, leukostasis was greatly exacerbated in retinal vessels of diabetics treated with PBM and SNPP, suggesting that HO-1 acts to mitigate the leukostasis in diabetes. Since (i) leukostasis involves binding of an activated leukocyte to an adhesion molecule (like ICAM-1), and (ii) SnPP did not alter the beneficial effect of PBM on ICAM-1 expression, these results imply that the effects of SnPP on leukostasis are mediated within the leukocytes. Likewise, SnPP inhibited the diabetes-induced reduction in HO-1 expression, apparently acting on a feed-back mechanism. Inhibition of HO-1 during PBM suppressed the diabetes-induced induction of iNOS expression better than that observed with PBM alone, suggesting that HO-1 activity somehow is involved in regulation of iNOS expression in diabetes.

Beneficial effects of PBM occur despite pigmentation, and are apparent when administered from either the onset of diabetes (prevention) [18] or after a period of untreated diabetes (intervention; present study and [17]). These findings provide evidence of beneficial actions of far-red light on important early changes of diabetic retinopathy, and show that PBM can inhibit development of diabetes-induced molecular abnormalities in the retina, as well as mitigating existing abnormalities. PBM is non-invasive, inexpensive, and easily to administer, and offers a non-pharmacologic approach to help inhibit lesions of diabetic retinopathy. Importantly, the beneficial effects are apparent with exposure to the light for only a few minutes per day.
